# Epidemiology of *Enterocytozoon bieneusi* Infection in Humans

**DOI:** 10.1155/2012/981424

**Published:** 2012-10-03

**Authors:** Olga Matos, Maria Luisa Lobo, Lihua Xiao

**Affiliations:** ^1^Grupo de Protozoários Oportunistas/VIH e Outros Protozoários, Unidade de Parasitologia Médica, CMDT, Instituto de Higiene e Medicina Tropical, Universidade Nova de Lisboa, 1349-008 Lisboa, Portugal; ^2^Division of Foodborne, Waterborne and Environmental Diseases, Centers for Disease Control and Prevention, Atlanta, GA, USA

## Abstract

A review was conducted to examine published works that focus on the complex epidemiology of *Enterocytozoon bieneusi* infection in humans. Studies on the prevalence of these emerging microsporidian pathogens in humans, in developed and developing countries, the different clinical spectra of *E. bieneusi* intestinal infection in children, in different settings, and the risk factors associated with *E. bieneusi* infection have been reviewed. This paper also analyses the impact of the recent application of PCR-based molecular methods for species-specific identification and genotype differentiation has had in increasing the knowledge of the molecular epidemiology of *E. bieneusi* in humans. The advances in the epidemiology of *E. bieneusi*, in the last two decades, emphasize the importance of epidemiological control and prevention of *E. bieneusi* infections, from both the veterinary and human medical perspectives.

## 1. Introduction

Microsporidia are a group of obligate intracellular eukaryotic parasites first identified as the cause of pébrine disease of silkworms in 1857 [1]. Over the past two decades, microsporidia have risen from obscure organisms to well-recognized human pathogens. Several genera and species of microsporidia were found in humans, and diagnosis and clinical management of microsporidiosis cases have improved significantly. Despite these scientific advances, the epidemiology of human microsporidiosis is still unclear. Most of what is now known about human microsporidiosis can be attributed to experience with patients infected with human immunodeficiency virus (HIV). Since 1985 and the first recognition of *Enterocytozoon bieneusi* as an acquired immunodeficiency syndrome (AIDS)-associated opportunistic pathogen [2], several hundred patients with chronic diarrhea attributed to this organism have been reported. Cases of intestinal microsporidiosis due to *E. bieneusi* have been increasingly reported in other immunocompromised individuals such as organ transplant recipients, as well as in travelers, children, and elderly [3–12]. Hepatobiliary and pulmonary involvement has also been observed in immunocompromised persons [13–15]. Some reports in humans suggested that this species can produce asymptomatic infections in immunocompromised and immunocompetent individuals [16–20]. In addition, *E. bieneusi* has been commonly identified in animals, especially mammals [21–32], and water, raising public health concerns about zoonotic and waterborne transmission of microsporidia [33–35].

## 2. Prevalence of *Enterocytozoon bieneusi* in Humans

*Enterocytozoon bieneusi* infections in humans are mainly detected by light microscopy of stained fecal smears, electron microscopy, and PCR-based methods. Therefore, infection rates are difficult to compare because of the considerable differences in the diagnostics methods employed, the specimens analyzed, the geographical locations, the patient groups, and patients characteristics (sex, age, socioeconomic conditions, immune status, and clinical features). 

In developed countries in North America, Europe, and Australia, studies involving HIV-seropositive persons with diarrhea reported rates between 2% and 78% [17, 36–45]. Lower infection rates (between 1.4% and 4.3%) were reported in HIV-seropositive persons without diarrhea [17, 39, 43, 45]. In one study of HIV-seronegative patients with diarrhea or pneumonia, spores of *E. bieneusi* were detected in 2.5% of urine, 11.5% of fecal, and 16.2% of sputum samples examined [46]. In majority of the studies performed in developed countries *E. bieneusi *was the species detected most often, followed by *E. intestinalis*; nevertheless, these findings are not in accordance with results of a study performed recently in Czech Republic, involving immunocompetent humans [20]. In this study, *E. bieneusi *was detected four times less frequently than *E. cuniculi*, and *E. intestinalis *was identified only sporadically. Also the majority of microsporidial infections (reaching 94%) were reported for nondiarrheal specimens [20]. Recently, a study was conducted in Portugal. It spans a period between 1999 and 2009, involving 856 patients (561 HIV-seropositive and 295-seronegative) and respective clinical data. The authors observed that *E. bieneusi* was more common in the HIV-seropositive group of patients (7.0%–39) than in the HIV-seronegative group (5.1%–15) [45]. 

In developing countries, *E. bieneusi* prevalence rates were reported between 2.5% and 51% in HIV-seropositive adult patients with diarrhea [47–60], and in 4.6% of patients without diarrhea [55]. Also in developing countries, in HIV-seronegative persons with and without diarrhea,* E. bieneusi* was detected in 5.35% to 58.1% of the fecal samples examined [19, 51, 53, 54, 61, 62]. In studies conducted in children, *E. bieneusi* prevalence ranged from 17.4% to 76.9% in HIV-seropositive children with diarrhea, and from 0.8% to 22.5% in the immunocompetent or apparently immunocompetent groups of children with or without diarrhea [18, 19, 30, 49, 63–68]. The recovery of spores of *E. bieneusi* in 0.8% of African children considered HIV seronegative, and in 5.9% of healthy orphans and 1.9% of child-care workers in Asia, indicated the presence of enteric carriage of infection in immunocompetent persons in tropical countries [49, 69].  Table 1 gives an overview of the studies conducted on the prevalence of *E. bieneusi* infection in humans, in various areas.

In industrialized nations, some studies reported *E. bieneusi* infection rates between 6.1 and 10% in travelers who suffered from self-limited diarrhea and returned from tropical destinations [6, 8]. In addition, a prevalence of 17.0% of *E. bieneusi* infection in elderly persons was observed, leading to the speculation that age-related diminishment of the immunity might predispose these persons to microsporidial infections [7].

A few prospective studies conducted in developed countries indicate that the prevalence of *E. bieneusi* in HIV-seropositive patients is progressively decreasing, probably due to the use of highly active antiretroviral therapy (HAART) [70, 71]. Similar observations are also made recently in some developing countries. In a study conducted in Brazil, comparing enteric parasitic infections in HIV/AIDS patients before and after the HAART, a statistically significant reduction in the prevalence of these infections was observed (63.9% versus 24%) [72]. Also in a 1-year longitudinal study of *E. bieneusi *infection in the same orphanage in Bangkok, Thailand, a decreasing pattern of prevalence, similar to the one observed for the incidence, was detected [19].

## 3. Patterns of *Enterocytozoon bieneusi *Intestinal Infection in Children

Microsporidia are ubiquitous in nature [73], and so it should come as no surprise that they can infect humans and cause clinical disease. *E. bieneusi* was the first microsporidia species observed by transmission electron microscopy in 1982 in villus epithelial cells in small intestinal biopsies from AIDS patients with diarrhea [74]. This species is closely linked with persistent diarrhea and wasting in adults who are HIV/AIDS positive; however, the outcome of follow-up studies involving children who are also HIV/AIDS-positive and severely malnourished children may be entirely different and warrants further study.

In the last decade, several studies reported the recovery of *E. bieneusi* spores from children' stools associated to different clinical spectra (symptomatic and asymptomatic infections in HIV-seropositive and seronegative children), in different settings (Table 1). 

In immunocompetent or apparently immunocompetent children, several cases of *E. bieneusi* infection have been described. In a study conducted in children considered HIV seronegative, attending two primary care centers in Niamey, Niger,* E. bieneusi *spores were recovered in 8 of 990 stool samples from these children (six males and two females, with ages ranging between 3 and 26 months). The methods used were chromotrope 2R staining and electronic microscopy for species confirmation. Seven of the *E. bieneusi* infected children were symptomatic, and one was asymptomatic. Three of the symptomatic ones presented other intestinal coinfections with *Giardia intestinalis* (one), *Entamoeba histolytica* (another), and *G. intestinalis* and *Trichomonas intestinalis* (the third child) and complaint of diarrhea. Due to these associations, the pathogenic role of microsporidia, in these children, is difficult to ascertain. Although, for the other four symptomatic children, whose stools were positive for *E. bieneusi*, this was the only enteropathogen identified; two had diarrhea only, one had diarrhea and vomiting, and the last one complaint of vomiting, dehydration, and fever without diarrhea [49]. Also a case of dual infection with *E. bieneusi* and *Cryptosporidium* was reported in a healthy three-year-old Turkish girl (HIV seronegative) living in Germany that accompanied her family on a vacation trip to Turkey, where they resided in a rural area. Two months later the child developed profuse watery, nonbloody diarrhea, and nausea with recurrent vomiting, weight loss, and serious dehydration. The diagnosis was performed using light microscopy and electronic microscopy for microsporidia species confirmation. The girl's diarrhea resolved without treatment after six weeks. As it happens in the above study the pathogenic role of microsporidia is difficult to ascertain in this child, due to the concomitant presence of *Cryptosporidium* in stools [10]. Another investigation was carried out to search for microsporidian spores in the stool specimens of 344 toddlers, essentially HIV seronegative, aged 1 to 24 months, and hospitalized at a pediatric institution in Tucumán, Argentina. They were classified in two groups: I, made up of 222 children suffering from severe diarrheas, and II by 122 affected by different pathologies, except gastroenteritis. The detection of microsporidia was done by light microscopy in smears of stained stools although the determination of the species involved was not done. Coproparasitological and coprobacteriological studies were also carried out and the nutritional status of each child was determined. In group I, microsporidia were found in 7.2% (16/222) of the children, 4/68 (5.9%) belonged to eutrophic children, and 12/137 (8.8%) to undernourished children; 8/16 positives were found to be related with other enteropathogenics. In group II, microsporidia were detected in 8.2% (10/122), 4/47 (8.5%) in eutrophic children, 4/54 (7.4%) in undernourished children, and without data in two cases; five out of 10 positives were related with other enteropatogenics. In this study, the occurrence of intestinal microsporidia was important and did not show significant differences between toddlers with or without diarrhea, eutrophic, or undernourished children [63]. An unusual *E. bieneusi* genotype (Peru 16), transmitted from guinea pigs, was found in a 2-year-old immunocompetent child complaining of diarrhea, in Peru. This child suffered weight loss that started almost concurrently with the episode of diarrhea at the beginning of this infection episode. This finding suggests that this species may cause short-lived gastrointestinal manifestation in immunocompetent persons [75]. Also, in a study conducted in Portugal, involving 295 HIV-seronegative patients with gastrointestinal complaints, of whom 18.6% (55) were children, it was observed that microsporidians were more common in children 18.2% (10/55), than in adults 6.3% (15/240), and this difference was statistically significant (*P* = 0.012). The age of the HIV-seronegative children infected ranged from 20 months to 8 years, with a median of 3 years. The percentage of infection by *E. bieneusi* was similar in both age groups, 5.5% for children against 5.0 % for the adults. In the same study, the overall percentage of HIV-seronegative patients with diarrhea (4.5%; 7), due to *E. bieneusi*, was slightly smaller than that of nondiarrheal (5.7%; 8) [45]. 

Studies have been conducted also in immunocompromised children, especially HIV-seropositive ones. In a prospective study conducted in Madrid, Spain, to determine the prevalence rates of microsporidiosis and other enteroparasites in 83 HIV-seropositive children treated in three hospitals of the city, *E. bieneusi* was found in 1.2% (one) of the children. Microsporidia spores were identified by staining methods and confirmed by PCR. The infected child was 10 years old and presented nonchronic diarrhea and a CD4 count of 298/mm^3^ [66]. In another investigation carried out in a day care treatment center and the National Hospital of Niamey, Niger, in HIV-seropositive patients, before the introduction of highly active antiretroviral therapy (HAART), one child out of a group of 24 patients, all adults, was positive for *E. bieneusi*, by real-time PCR. However, no other clinical data were provided in the study [76]. A recent study conducted in, Lisbon, Portugal, involved 126 HIV-seropositive children, in a total of 561 HIV-seropositive patients with gastrointestinal complaints of whom 18.6% (55) were children, it was observed that microsporidians were more common in children 19.0% (24), than in adults 12.4% (54). The percentage of infection by *E. bieneusi* was higher in children (10.3 %; 13) than in adults (6.0%; 26), but this difference was not statistically significant. In the same study, the overall percentage of HIV-seropositive patients with diarrhea (8.6%; 29/339), due to *E. bieneusi*, was lower than that of nondiarrheal (4.5%; 10/222). Also here the difference was not statistically significant [45]. 

Also studies have been carrying out in institutionalized and inpatient children, independent of the HIV status. A study was undertaken in Dar es Salaam, Tanzania, to relate the protozoan infections by *Cryptosporidium*, microsporidia, and *Cyclospora* with the clinical features and HIV status of children, aged 15–60 months, and adults hospitalized with diarrhea. Included in the study there were two groups of inpatient symptomatic children: children with chronic diarrhea (of whom 23 of 59 were HIV-seropositive) and children with acute diarrhea (of whom 15 of 55 were HIV seropositive). Microsporidia spores were identified by staining methods and the species confirmed by transmission electron microscopy.* Enterocytozoon *was identified in specimens from 2/59 children with chronic diarrhea (one HIV seropositive) and in 0/55 children with acute diarrhea [77]. Another study designed in Bangkok, Thailand, investigated the prevalence and clinical features of intestinal microsporidiosis in hospitalized HIV-seropositive and seronegative children with diarrhea. Of the 95 HIV-seropositive and 87 seronegative children, 25.3% (24) and 14.9% (13), respectively, were diagnosed with intestinal microsporidiosis. Species identification of microsporidia spores by transmission electron microscopy demonstrated *E. bieneusi* in five cases. *Cryptosporidium parvum* was a common coinfective parasite. Malnutrition was commoner in the HIV-seropositive group (79.2% versus 23.1%; *P *= 0.003). This study indicated that intestinal microsporidiosis was an important disease in both HIV-seropositive and -seronegative Thai children with diarrhea [65]. In a cross-sectional, case-control study, involving children aged <60 months, with undetermined HIV status, undertaken in Kampala, Uganda, using genetic tools, a total of 17.4% of 1,779 children with diarrhea, attending a hospital, were infected with *E. bieneusi* compared with 16.8% of 667 control children (without diarrhea). There was no significant relationship between infection with *E. bieneusi* and stunting, being underweight, wasting, or acute diarrhea. The authors observed that children with a high rate of excretion of spores were more likely to have diarrhea of longer duration (*P *< 0.001), which they hypothesized could suggest an association with more prolonged illness. It was concluded that *E. bieneusi* was widespread among children 3–36 months of age in Uganda, and that there was no clear association of *E. bieneusi* with poor nutrition or diarrhea [18]. The same authors, in a following study involving 243 children, aged <60 months with persistent diarrhea (>14 days), admitted to the same hospital in Kampala, Uganda, using microscopy and PCR methods, screened for the presence of *E. bieneusi* and *Cryptosporidium* in the children' stools [64]. They found that 32.9% (80) of them were excreting *E. bieneusi*, and 31.3% (76) were excreting *Cryptosporidium*. Ninety-one of the 243 children were HIV seropositive, of whom 76.9% (70) had *E. bieneusi*, versus 6.6% (10) of the 152 HIV-seronegative (odds ratio = 47.33; 95% CI = 19.88 to 115.97), while 73.6% (67) had *Cryptosporidium*, versus 5.9% (9) without HIV (odds ratio = 44.36; 95% CI = 18.39 to 110.40). Children with counts <25% CD4 cells were more likely to have either *E. bieneusi* (odds ratio = 7.42; 95% CI = 3.77 to 14.69) or *Cryptosporidium *(odds ratio = 6.45; 95% CI = 3.28 to 12.76) than those with higher CD4 percentages. However, only HIV status was independently associated with either *E. bieneusi *or* Cryptosporidium*. Among the 243 children with persistent diarrhea, 27.8% (67) were infected with both enteric pathogens, with HIV being the only independent predictor of coinfection. Finally, some 81% of HIV-seropositive children with persistent diarrhea excreted one or both organisms, compared with only 10% of children with persistent diarrhea testing negative for HIV. As in their previous study on *E. bieneusi *[18] there was no significant association between infection in this subpopulation and malnutrition even after controlling for HIV status and CD4 counts/percentages. Once more authors concluded that *E. bieneusi *as well as *Cryptosporidium *were highly prevalent in Ugandan children, especially those immunocompromised by HIV/AIDS [64]. Also a study was conducted among hospital patients and school children in Vhembe district, Limpopo Province, South Africa, for microsporidia detection using PCR-RFLP and staining methods. In the age group between 0 and 9 years, *E. bieneusi* was found in 50 children (six HIV seropositive) attending the hospital, of whom 80% (40/50) complaint of diarrhea, and in nine school children (apparently healthy) in the same age group, of whom 33% (3/9) had diarrhea [54]. In a cross-sectional study of intestinal microsporidiosis conducted, this time in Bangkok, Thailand, in children who lived in an orphanage, stool samples were collected from 290 orphans (39 HIV seropositive and 251 HIV seronegative). Stool analysis was made under light microscopy, and confirmation of species was performed using transmission electron microscopy. Of the 290 children, 4.1% (12) were positive for *E. bieneusi* [5]. Taking into consideration the results obtained in this study, the same authors conducted a 1-year-longitudinal study of *E. bieneusi *infection in the same orphanage. A total of 540 orphans and 81 child-care workers were enrolled in the study. Using microscopy and PCR methods these authors found that 13.9% (75) of the orphans had *E. bieneusi* spores in fecal samples. Only 28.6% (2/7) of the diarrheic children were infected by *E. bieneusi*. In this study *E. bieneusi *infection was significantly prevalent in children between 1 and 3 years of age with a lower incidence in the older age group, which may reflect the development of protective immunity. Nine orphans (three HIV seropositive) were reinfected (4-month intervals between two positive PCRs) in the study. Thus, the authors hypothesized that protective immunity might not be fully developed after an infection in some children since reinfection occurred [19]. 

Asymptomatic infections with *E. bieneusi* have been reported in both HIV-seropositive and -seronegative adults although in children the asymptomatic cases were described, so far, only in HIV-seronegative children. In a study performed in Tanzania, in four of 20 HIV-seronegative children admitted to hospital due to acute trauma or elective surgery, with no history of diarrhea in the previous 3 months, *E. bieneusi* spores were identified by staining methods and the species confirmed by transmission electron microscopy. All four asymptomatic children were underweight (*P *< 0.01). In the cross-sectional study of parasitic infection conducted in Bangkok, Thailand, in orphan children, spores of *E. bieneusi* were detected in stool specimens, by a staining method and confirmed by transmission electron microscopy, in 5.9% (13/221) of the HIV-seronegative children who had neither current illness nor history of gastrointestinal symptoms in the previous 1 month [69]. Also in Kampala, Uganda, *E. bieneusi* was detected by molecular methods in stool samples of 112 children, with undetermined HIV status, without diarrhea [18]. In the study conducted in Limpopo Province, South Africa, *E. bieneusi* asymptomatic infections were detected in 20% (10/50) of the children aged 0 to 9 years, observed in the hospital, and in 67% (6/9) of the school children enrolled in the study, by PCR methodology [54]. In another study, in Cameroon, *E. bieneusi *was, once more, detected in stools samples from healthy persons without symptoms, including teenagers (12–19 years old) and children in the age group between 0 and 11 years. The percentage of infection increased with age; however, children had the highest parasite loads [53]. 

The pathogenicity of microsporidia is still not clearly defined, and the mechanism by which they induce diarrhea has not been determined. Some authors found that even though HIV-positive patients infected by *E. bieneusi *had more diarrhea than those noninfected, they in fact had less inflammation than the noninfected HIV-positive individuals, demonstrated by a lactoferrin test, even though some genotypes of *E. bieneusi *can cause chronic diarrhea [48, 54]. 

*Enterocytozoon bieneusi* can be an important cause of persistent diarrhea, intestinal malabsorption, and wasting in HIV-positive adults. Mucosal damage associated with microsporidiosis is more extensive than that related to other opportunistic intestinal infections and leads to substantial malabsorption of vitamins, micronutrients, carbohydrates, and fats [71, 78, 79]. Although microsporidiosis is common in children <5 years of age, particularly those who live in developing countries or who are HIV-seropositive, the effects of infection on nutritional health of these vulnerable populations were not well documented until recently [18, 64, 65, 80]. In a study conducted in Uganda, in children ≤60 months of age with persistent diarrhea *E. bieneusi* was associated with lower rates of weight gain. This relationship remained after controlling for HIV and concurrent cryptosporidiosis. Children with microsporidiosis were predicted to weigh 1.3 kg less than children without microsporidiosis at 5 years of age [81].

Diarrhea and malabsorption seem to be the most common clinical problems associated with microsporidian infections, namely, *E. bieneusi*, reported in the literature [14, 46, 82]. However in some studies there were no statistically significant associations between the presence of microsporidia, namely *E. bieneusi*, in faecal specimens and patients with diarrhea [45, 55, 83–85]. Several studies suggest that *E. bieneusi* infection can remain asymptomatic suggesting that some persons may be asymptomatic carriers of these microorganisms [19, 49, 69, 77].

## 4. Risk Factors Associated with *Enterocytozoon bieneusi* Infection

Since the identification of *E. bieneusi* as a pathogen in humans, it has been shown to be a major cause of chronic diarrhea in HIV-seropositive persons. Some studies have tried to identify risk factors for *E. bieneusi* infection in humans (Table 2). Immunodeficiency, especially that associated with HIV/AIDS, low CD4+ T cells count (≤50 cells per microliter blood), and younger age are all considered risk factor for intestinal microsporidiosis due to *E. bieneusi* [18, 19, 45, 64, 86, 87]. In Ethiopia, in a study conducted in 214 HIV-seropositive and 29 HIV-seronegative patients with diarrhea, microsporidial parasites (*E. bieneusi* and *E. intestinalis*) were detected only in HIV-seropositive patients (39). Among the patients studied 92.3% had diarrhea for over four weeks, and 94.9% had weight loss of more than 10% [82]. Also in a recent study performed in Russia, involving 159 HIV-seropositive patients, a statistically significant association was observed between the microsporidia positive patients and weight loss of >10% of the baseline (63%; 19/30) [87]. In a 10-year study, performed in Portugal, involved 856 (675 adults and 181 children) HIV-seropositive and -seronegative patients with gastrointestinal complaints, an immunosuppressive condition and youth (children) were the risk factors observed for microsporidian infection [45].

Other risk factors associated with intestinal microsporidiosis, in general, or particularly to *E. bieneusi* infection have been reported. A US study of HIV-seropositive patients with diarrhea showed significant association of intestinal microsporidiosis with contact with horses, having been stung by a bee, hornet, or a wasp, and having used injection drugs. The use of hot tub or spa and occupational contact with water also reached statistical significance in this study [86]. Also person-to-person transmission has been suggested in an orphanage, where a multivariate analysis showed that orphans who were 12–23 months old, girls, and living in one particular house were independently associated with *E. bieneusi* infection. All infected children presented the same *E. bieneusi* genotype in stools [5, 19]. Another study in Zimbabwe showed association of *E. bieneusi* infection in HIV-seropositive patients with living in rural areas, consumption of nonpiped water, contact with cow dung, and contact with a person with diarrhea [50]. These authors postulated the reasons for the unclear association between injection drug use and enteric microsporidiosis stating that injection drug users were as likely or more likely to contact potential infectious sources, such as contaminated water. Contact with water is an important risk factor consistently associated with *E. bieneusi* infection in epidemiologic studies, and *E. bieneusi* spores have been detected in ground, surface, ditch, and crop irrigation water sources [33, 105–107, 132]. However, no significant seasonal variation has been detected in the prevalence of intestinal microsporidiosis in HIV-seropositive patients [70]. In a study of HIV/AIDS patients performed in Peru, risk factors for *E. bieneusi* infection differed by genotype. Infection with genotype Peru-1, one of the more common genotypes found, was associated with contact with duck or chicken droppings and lack of running water, flush toilet, or garbage collection. In contrast, genotypes Peru-2 to Peru-11 were associated with watermelon consumption [48].

*E. bieneusi* has been able to cause clinical disease in immunocompromised and immunocompetent persons with different patterns, and the risk factors associated with these pathologies are partially known as a result of improvement in genetic typing and molecular epidemiology. 

## 5. Molecular Epidemiology of* Enterocytozoon bieneusi* in Humans

The combination of epidemiologic studies with genotyping techniques has contributed to a better understanding of the characteristics of microsporidia that infect humans and of its reservoirs and transmission patterns. Before the introduction of the molecular tools, the characterization of this important group of parasites relied exclusively on electron microscopic observations of the morphological differences in spores and/or endogenous developmental stages and host occurrence. The recent application of PCR-based molecular methods for species-specific identification and genotype differentiation of microsporidia has increased research interests in this group of microorganisms. The enhanced diagnostic capacity obtained with the molecular methods has improved the identification of human cases of microsporidiosis due to *E. bieneusi*, not only in developed countries [6, 7, 17, 20, 30, 38, 45, 62, 66, 81, 87, 92, 108, 112, 115], but also, and especially, in developing countries where *E. bieneusi* may be a public health problem due to the magnitude of the HIV pandemic and poor sanitary conditions [18, 19, 30, 47, 48, 50–52, 54–56, 62, 64, 68, 88, 89, 99, 102]. 

*Enterocytozoon bieneusi* is a complex species with multiple genotypes and diverse hosts range and pathogenicity. Since different strains cannot be discriminated morphologically, typing of this species relies on molecular methods [22]. PCR analysis of the internal transcribed spacer (ITS) of the rRNA gene, a hypervariable sequence with about 243 bp long, followed by sequencing of the PCR products and comparison of obtained ITS sequences with those in the databases, is the standard method for genotyping *E. bieneusi *isolates from humans and animals. For several years these were the only genetic markers available [22, 56, 104, 133–136]. A few researchers genotyped *E. bieneusi* based on the analysis of the ITS by PCR-restriction fragment length polymorphism (RFLP) [92, 109]. And recently, two groups of researchers reported the analysis of the GenBank *E. bieneusi* ITS sequence collection, using statistical methods, with the aim to obtain information about diversity, transmission, and evolution of this species [135, 136]. These studies have identified the presence of host-adapted *E. bieneusi* genotypes in a variety of domestic animals and wild mammals, as well as a large group of *E. bieneusi* genotypes that do not appear to have host specificity [24, 25, 135, 136]. These latter genotypes, considered zoonotic, are responsible for most human infections. *E. bieneusi* genotypes in humans have been shown to differ from each other in virulence and geographic distribution [48, 136]. Other authors, taking advantage of the current genome sequence surveys of *E. bieneusi*, identified four polymorphic microsatellite and minisatellite markers. Together with the ITS, they became part of a multilocus sequence typing (MLST) technique developed for genotyping *E. bieneusi* that could be useful in epidemiologic investigations of *E. bieneusi *transmission, especially those concerning the public health significance of parasites of animal origin [137]. The observations, obtained with the study of the ITS locus, were also corroborated with the phylogenetic analysis of the polymorphic microsatellite and minisatellite markers, identified by Feng and collaborators in the *E. bieneusi *genome. These authors observed the formation of two large groups of *E. bieneusi*: zoonotic genotypes and host-adapted genotypes [137].

Actually, more than 90 different genotypes of *E. bieneusi, *identified by ITS sequence, have been described in humans and animals, and some genotypes have multiple names that have been reviewed in Satín and Fayer, 2011 [138] who tabulated all the existing synonyms for the first time and proposed a standardized nomenclature for *E.bieneusi* genotypes based on the ITS sequences. About 34 of these genotypes have been found only in humans [5, 18, 24, 48, 75, 76, 92, 94, 99, 109, 112, 114, 115] (Table 3), and 11 genotypes have been reported both in humans and animals [18, 21, 24–29, 48, 55, 56, 75, 76, 92, 95, 99, 102, 109, 111–114, 116, 118–127, 129, 139] (Table 4). All these observations were made in populations from different geographic regions, involving all continents. 

In studies conducted in France involving HIV seropositive and seronegative patients infected with *E. bieneusi*, Liguory and collaborators found five genotypes (Types I to V) [109, 112]. Type I, synonym of genotype B, was the most prevalent genotype (75%). It was followed by Type II, designated as genotype C (10%). Genotypes B (Type I) and C (Type II) were also found in other populations [94, 95, 99, 111, 112, 115]. Genotype B (Type I) even showed predisposition toward HIV/AIDS patients [112] and was the only genotype found in a study performed in HIV-seropositive patients in Australia [115]. Type IV, also named genotype K/Peru2/PtEbIII/BEB5/BEB5-var/CMITS1, was rare (only in one human isolate) [92]. In contrast, Type IV (genotype K) was the only genotype found in a population in Cameroon [55]. Moreover, this genotype was also found in humans in other regions [18, 48, 56, 75, 76, 99, 111, 112] and in various animals [25–27, 122–125]. Type V was found only in one HIV-seropositive person.

In Lima, Peru, in a population of 2,672 HIV/AIDS patients, 11 genotypes were identified (Peru-1 to Peru-11). The most prevalent genotypes were Peru-1 (39%), synonym of genotype A, Peru-2 (19%), synonym of Type IV, and Peru-9 (10%), also named genotype D; the remaining eight genotypes were found in the population at much lower frequencies (1–9%) [48, 56, 75]. Some of these genotypes were also found in animals, including genotype A (Peru-1), Type IV (Peru-2), Peru-4, also named genotype EbpC/E/WL13/WL17, to Peru-6, also named PtEb1/PtEbVII, Peru-7, D (Peru-9), Peru-10 and Peru-11, also named Peru-12 [21, 24–28, 32, 55, 75, 95, 114, 116, 117, 121–126]. Three genotypes (A/Peru-1, Peru-7, and Peru-11), found until recently only in humans, were also identified in animals [32, 117]. Both genotypes D and Type IV (reported as genotype K) were the most prevalent among patient groups from Malawi and The Netherlands [112]. Genotype D that was commonly detected in HIV-seropositive patients was also found in HIV-seronegative individuals, confirming its widespread nature [56, 99, 101, 102, 111, 113]. In a study conducted in renal transplant recipients in Spain, genotype D was the only *E. bieneusi* genotype found [108].

Also in Peru, in a prospective pediatric-cohort study of enteric parasites, *E. bieneusi* infection was identified in 31 of 388 (8%) children [75]. Thirty of these children had infections with genotypes Peru-1 (synonym of A) through Peru-15 and Peru-17, and one child was infected with genotype Peru-16. Fecal samples of all animals in the household of child infected with Peru-16 were also analyzed including guinea pigs, chickens, dogs, and cats. Peru-16 was identified in seven of the eight guinea pigs studied, and all the other animals were negative for microsporidia. This genotype was genetically very different from other known *E. bieneusi *genotypes in humans and was placed, in an independent clade, outside the cluster of genotypes considered to have broad host specificity in a phylogenetic analysis of the ITS sequences. The finding of genotype Peru-16 in guinea pigs from unrelated households, and the close contacts between the study child and infected guinea pigs, all strongly suggested that the child probably acquired the infection from the guinea pigs in the household [75]. This finding indicated that even some *E. bieneusi *genotypes that are apparently unique in some animals have zoonotic potential [119]. One of the genotypes, Peru-14, also named genotype WL15/WL16, was also found in animals [24].

Genotype UG2145, identified for the first time in Uganda, was the only *E. bieneusi* detected in a population of 1,779 children with diarrhea [18].

Some genotypes like genotypes B and C are considered host specific because they were exclusively found in humans. However, it must be kept in mind that they differ in very few base pairs from other sequences commonly found in other hosts. For instance, genotype type IV differs from B in 3 bp, and from A in just 1 bp. In contrast, some other genotypes have a zoonotic potential (such as genotypes D and Type IV, and more recently genotype A) because they have been seen in various animals [18, 24–27, 29, 48, 75, 76, 95, 111, 112, 116, 118, 119, 122–124, 126, 127, 129, 139]. 

Breton and collaborators revealed for the first time the presence of genotypes A and B in Africa [99]. These genotypes were also reported from HIV-seropositive and HIV-seronegative populations in Europe [92, 94, 109, 111, 112, 121], Peru [56, 75], Thailand [5, 114], and Niger [76]. In Breton and collaborators study, genotype D was found in one HIV-seropositive patient in Gabon, and it was identified for the first time in isolates from three HIV-seronegative individuals in Cameroon [99]. It is considered a genotype with a broad host and geographic range, having been found in several countries in Europe [26, 111, 112, 127], USA [24, 119], Peru [48, 56, 75], Malawi [112], Thailand [5, 102], Vietnam [76], and Korea [124, 129]. Three new genotypes CAF1, CAF2, and CAF3 that are close relatives of genotypes E, Type IV, and D, respectively, were found for the first time in the HIV-seropositive patients from Gabon, with 10 out of 15 isolates. CAF1 was also found in pigs in Korea [120]. Another new genotype, CAF4, a highly divergent genotype reported from humans, was equally present in HIV-seropositive patients in Gabon and HIV-seronegative individuals in Cameroon. Its high frequency (25%) in both countries may indicate that this genotype is common in Central Africa [99]. 

Ten Hove and collaborators found nine new genotypes: six (S1-S6) were from Malawi, and three (S7-S9) were from The Netherlands [112]. Genotype S7 showed no linkage to any of the described genotypes and seems to be more related to the genotypes isolated from cattle. The patient (HIV infection was considered unlikely) infected with this *E. bieneusi* genotype was a middle-age man presenting severe diarrhea and rectal bleeding. Two other new unnamed genotypes were detected in this study, AF502396 and AY371283, actually named as UE2145 and Peru-8, respectively.

In a study conducted in Changchun City, China, involving 40 fecal samples from diarrheal children and 180 fecal samples from animals (61 from pigs, 26 from dogs and 93 from cows), all collected in the same area around the city, Zhang and collaborators found two previously reported genotypes (genotypes I and J), and 10 new genotypes (CHN1-10) [30]. Genotypes I (also named BEB2/CEbE) and J (also named BEB1/CEbB, PtEb X), originally detected in cattle [25], were identified in both cow and human samples in this study. Both genotypes coinfected with other genotypes in all positive samples. Of the novel genotypes, CHN1, which was detected in five children, nine cows, and four pigs, was the most common genotype found in this study. Genotype CHN2 was found only in human samples. Genotypes CHN3 and CHN4 were also found in both human and cow samples, and genotypes CHN5-10 were only detected in animals. The ITS sequences of the 10 new genotypes were highly homologous to those of genotypes I, J, Type IV, and G published earlier [25, 75, 101, 109, 129]. Genotypes CHN1, CHN2, and CHN3 differed from J by one to four positions, while genotype CHN4 is 2 bp shorter than type IV. Due to the high level of similarity of the sequences, no obvious clusters related to host preference were observed [30]. 

In a study that investigated the occurrence and prevalences of the microsporidial species *Enterocytozoon bieneusi *and *Encephalitozoon *spp. in 382 immunocompetent humans in the Czech Republic the sequence analyses of *E. bieneusi*-positive samples revealed seven different genotypes [20]. For the first time, after so many studies performed in humans, in different geographic areas, genotypes that were previously reported only from cattle (BEB4 and EbpA) and pigs (PigEBITS5, EbpA, and BFRmr2) were identified in humans, and three new genotypes were detected (*E. bieneusi *CZ1 to CZ3). The most prevalent *E. bieneusi *genotypes were EbpA (in 10 individuals), CZ3 (in 4 individuals), and PigEBITS5 (in three individuals), followed by BFRmr2 and BEB4 (each in two individuals). The CZ1 and CZ2 genotypes were identified only in single individuals. In the same study *E. cuniculi *genotype II was also found in humans. These data suggest that isolates of this group are able to switch hosts from animals to humans and should be regarded as isolates with high zoonotic potential. The high prevalences of microsporidia infection surprisingly found in different populations of immunocompetent humans in this study raise the question of whether these findings represent true infection resulting in shedding of parasites or ingested parasites that just passed through the gastrointestinal tract without establishing an infection. However, the chance of detecting the temporary passage of spores is quite low in the number of individuals sampled analyzed in this study [20].

Several studies indicate that the distribution of genotypes of *E. bieneusi *can vary by geographical locations [95], and it has been recently proposed that predominant genotypes in different geographical sites could be related to distinct sources of transmission [5]. In a comparative study of the molecular epidemiology of microsporidiosis among HIV-seropositive patients in two separate geographical areas, Niamey, Niger and Hanoi, Vietnam, interesting results were obtained. In Hanoi, two zoonotic genotypes D and E were identified [76]. Both genotypes have been previously recovered in both humans and animals [21, 24, 26, 56]. These genotypes were also predominant in a study conducted in Thailand in HIV-seropositive patients [113]. Thus, there is a potential zoonotic transmission of *E. bieneusi *in Thailand and Vietnam. Zoonotic transmission can be from direct exposure to animals or by contamination of surface water by discharged domestic wastewater or animals. On the contrary, the Niamey data highlighted a higher frequency of genotypes known to be human specific (genotype A), similar to results obtained in children from an orphanage in Thailand [5]. These results suggest that transmission of microsporidiosis due to some *E. bieneusi* genotypes could occur through person-to-person contact. This mode of transmission could be facilitated by chronic carriage of *E. bieneusi*, as described in previous studies [49, 140]. 

All these findings suggest different transmission modes of *E. bieneusi *in diverse geographic regions and in special groups in the same region. However, further studies with larger number of *E. bieneusi* samples are needed to confirm these findings. 

## 6. Conclusion

Despite recent advances in the understanding and diagnosis of *E. bieneusi*, emerging microsporidian pathogens, more research is necessary to elucidate their complex epidemiology. In fact, studies that reflect true human infecting *E. bieneusi* prevalence are still insufficient. A few prospective studies conducted in developed countries indicate that the prevalence of *E. bieneusi* in HIV-seropositive patients is progressively decreasing, probably due to the use of HAART. Similar observations have also been made recently in some developing countries. The recovery of spores of *E. bieneusi* in fecal samples of children considered HIV-seronegative, and in healthy ones, indicated the presence of enteric carriage of infection in immunocompetent persons in tropical countries, but also in other regions of the globe. Also, the finding of asymptomatic chronic carriers of *E. bieneusi* emphasizes their importance in the transmission cycle, warning for the potential risk of reactivation of latent infection in cases of immunosuppression causing life-threatening disease in these individuals.

The pathogenicity of microsporidia is still not clearly defined, and the mechanism by which they induce diarrhea has not been determined. Even though some genotypes of *E. bieneusi *can cause chronic diarrhea and wasting syndrome in AIDS patients once the CD4+ T-lymphocyte count drops below 100 cells/mm^3^.

The recent application of PCR-based molecular methods to *E. bieneusi* identification and characterization has led to more reliable results compared with prevalence rates determined by light microscopy of stained biological smears, to better study the risk factors associated with these pathologies, to study the distribution of genotypes of *E. bieneusi *by geographical location, to improve source tracking, and to calculate the host range and pathogenic potential of an isolate. All typing studies performed until now were based on the analysis of the ITS sequences, the only molecular marker existent until recently. These studies identified the presence of host-adapted *E. bieneusi* genotypes in several animals, and *E. bieneusi* genotypes with no host specificity, which are considered zoonotic. These zoonotic genotypes are responsible for most human infections. Some studies suggest that transmission of microsporidiosis due to some *E. bieneusi* genotypes could occur through person-to-person contact. This mode of transmission could be facilitated by chronic carriage of *E. bieneusi*, as described in some studies. Besides, several microsporidian species that infect humans have been identified in animals, especially mammals, raising public health concerns about zoonotic and waterborne transmission of microsporidia. In addition, some studies indicate that even some *E. bieneusi *genotypes, which are apparently unique in some animals, have zoonotic potential. Other studies indicate that the distribution of genotypes of *E. bieneusi *can vary by geographical locations and virulence.

Despite of the great genetic variation of *E. bieneusi* observed worldwide with the analyses of the ITS region of the rRNA gene, studies with additional independent markers for *E. bieneusi *are highly desirable in order to clarify the genetic structure of the parasite's populations. The discrimination among strains of *E. bieneusi* is very important, not only for the clarification of the reservoirs and of the transmission modes of this pathogen but also to help to explain its variations in pathogenicity that cannot be answered with the study of a single locus.

## Figures and Tables

**Table 1:** Studies on prevalence of *Enterocytozoon bieneusi* infection in humans.

Continent	Country	Patient source	Major clinical presentation	Specimens analyzed	Diagnostic method	Prevalence (%) of *E*. *bieneusi* infection	Reference
	USA	HIV+	Chronic diarrhea	Intestinal biopsies	LM, TEM	30% (20/67)	[16]
	USA	HIV+	Chronic diarrhea and other gastrointestinal complaints	Duodenal biopsies	TEM	33% (18/55) with chronic diarrhea; 25% (13/51) without chronic diarrhea	[83]
	USA	HIV+	Chronic diarrhea	Fecal samples, intestinal biopsies	LM, confirmed by EM	78% (14/18)	[36]
	USA	HIV+	Diarrhea, other complaints	Duodenal biopsies	PCR, confirmed by TEM	44% (30/68) with diarrhea; 2.3% (1/43) without diarrhea	[17]
	USA	HIV+	Diarrhea	Fecal samples	LM	8.8% (137/1,557) in 1993; 9.7% (193/1,991) in 1994; 6.6% (155/2,346) in 1995; 2.9% (73/2,545) in 1996	[70]
America	Peru	HIV+	Diarrhea	Fecal samples	LM, confirmed by PCR	3.9% (105/2,672)	[56]
	Peru	HIV+	Diarrhea and other gastrointestinal complaints	Fecal samples	LM, confirmed by PCR	2.2% (56/2506)	[48]
	Peru	Children apparently immunocompetent	Diarrhea, weight loss	Fecal samples	LM, confirmed by PCR, sequence analysis	8% (31/388)	[75]
	Colombia	HIV+	Diarrhea	Fecal samples	LM, PCR	2.9% (3/103)	[88]
	Brasil	HIV+	Chronic diarrhea	Fecal samples, intestinal biopsies	LM, TEM or PCR	27.5% (11/40)	[89]
	Cuba	HIV+ adults; HIV– adults	Diarrhea, other complaints	Fecal samples	LM	(0/67) in HIV+; (0/136) in HIV–	[90]

	Portugal	HIV+	Diarrhea	Fecal samples	LM, PCR	29% (20/69)	[38]
	Portugal	HIV+, HIV−; Adults and children	Gastrointestinal complaints	Fecal samples	PCR	6.3% (54/856)	[45]
	Spain	HIV+ children	58% with diarrhea	Fecal samples	LM, confirmed by PCR	1.2% (1/83); 2% (1/48) with diarrhea	[76]
	Spain	Returning travelers from tropical countries	Travelers' diarrhea	Fecal samples	LM, confirmed by PCR	10% (4/40)	[6]
	Spain	HIV– elderly	Diarrhea	Fecal samples	LM, confirmed by PCR	17.0% (8/47)	[7]
	Spain	Immunocompetent	Diarrhea, pneumonia	Fecal, urine and sputum samples	LM, PCR, hybridization	11.54% (18/156) in feces; 16.22% (6/37) in sputa; 2.5% (1/40) in urine	[46]
	France	HIV+	Chronic diarrhea	Fecal samples and intestinal biopsies	LM, partially confirmed by EM	50% (9/18)	[41]
	France	HIV+	Diarrhea	Duodenal biopsies	LM	1.6% (1/61)	[37]
	France	HIV+	Chronic diarrhea	Fecal samples	LM	24% (11/46)	[91]
	France	HIV+; HIV–	Diarrhea	Fecal samples	LM, partially confirmed by EM, PCR-RFLP	88 HIV+ *E*. *bieneusi* infected; 12 HIV– (10 immunocompromised and 2 immunocompetent) *E*. *bieneusi* infected*. *	[92]
	Germany	HIV+	Diarrhea	Intestinal biopsies	PCR	21.7% (10/46)	[93]
	Germany	HIV+	Chronic diarrhea	Fecal samples	LM, PCR, sequence analysis	12 fecal samples from 8 patients	[94]
	Germany	HIV+	Diarrhea, other complaints	Fecal samples	LM	36% (18/50) with diarrhea; 4.3% (2/47) without diarrhea	[43]
Europe	Germany	Human patients	Diarrhea	Fecal samples	PCR, sequence analysis	7.7% (2/26)	[95]
	Germany	Returning travelers from tropical countries	Travelers' diarrhea	Fecal samples	LM, PCR	0.7% (1/148); 4.7% (7/148)	[8]
	Switzerland	HIV+	Diarrhea, other gastrointestinal complaints	Fecal samples	LM, partially confirmed by EM and PCR	12.7% (20/164) in 1992–94; 5.8% (9/156) in 1994–96; 0.4% (4/949) without diarrhea	[71]
	Holland	HIV+	Diarrhea	Fecal samples	LM, confirmed by EM	7.7% (11/143)	[96]
	UK	HIV+	Diarrhea	Fecal samples, intestinal biopsies	LM, EM, confirmed by TEM	14.3% (9/63) in fecal samples; 7.9% (3/38) in biopsies	[40]
	UK	HIV+	Gastrointestinal complaints	Fecal samples	LM, PCR, qPCR, TEM	8.3% (14/168)	[97]
	Italy	HIV+	Chronic diarrhea	Intestinal biopsies	LM, TEM	4.2% (3/72)	[44]
	Czech Republic	Asymptomatic healthy people	NA	Fecal samples	PCR	6.0% (23/382);	[20]
	Russia	HIV+, adults	Diarrhea	Fecal samples	PCR	1.3% (2/159)	[87]
	Russia	HIV/AIDS patients	NA	Serum	IFAT	13.0% (6/46)	[98]

	Tunisia	HIV+HIV−	Diarrhea, other gastrointestinal complaints	Fecal samples	PCR	20% (7/35) in HIV+ 5.35% (3/56) in HIV−	[62]
	Mali	HIV+, and HIV–	80% with chronic diarrhea	Fecal samples	LM, partially confirmed by TEM	32% (28/88) in HIV+; 27% (3/11) in HIV–	[51]
	Mali	HIV+ adults;HIV– children	Diarrhea	Fecal samples	LM, IFAT, PCR	13.1% (8/61) in HIV+; (0/71) in HIV–	[47]
	Niger	HIV+ (227 adults, 1 child)	NA	Fecal samples	LM, RT-PCR, sequence analysis	10.5% (24/228), all but one were adults	[76]
	Niger	HIV+ adults; HIV– children	Diarrhea, other gastrointestinal complaints	Fecal samples	LM, partially confirmed by EM	In adults 7% (4/60) with diarrhea; in children 1% (6/593) with diarrhea, 0.5% (2/397) without diarrhea	[49]
	Nigeria	HIV− children	Diarrheic and nondiarrheic children	Fecal samples	PCR, sequence analysis	9.3% (4/43)	[67]
	Nigeria	HIV+ children	Diarrheic and nondiarrheic children	Fecal samples	LM, IFAT, PCR, sequence analysis	2.6% (193)	[68]
	Cameroon	HIV+	Diarrhea, other complaints	Fecal samples	LM, PCR-RFLP	6.5% (3/46) with diarrhea; 4.6% (5/108) without diarrhea	[55]
	Cameroon	Villagers <1 to 80 years;	NA	Fecal samples	IFAT-MAb, confirmed by PCR, sequence analysis	2.9% (22/758) in HIV– 0.5% (4/758) HIV+	[99]
	Cameroon	HIV+ with TB; HIV– with TB; apparently immunocompetent persons	Diarrheic and asymptomatic persons	Fecal samples	LM, IFAT-MAb	35.7% (10/28) in HIV+ with TB; 24,0% (6/25) in HIV– with TB; 67.5% (85/126) in immunocompetent persons	[53]
Africa	Gabon	HIV+ > 16 years	Diarrhea, other gastrointestinal complaints	Fecal samples	IFAT-MAb, confirmed by PCR, sequence analysis	3.0% (25/822)	[99]
	Democratic Republic of the Congo	AIDS patients, >15 years	Diarrhea, other gastrointestinal complaints	Fecal samples	PCR	5.1% (9/175) in hospital patients	[60]
	Uganda	Children (immune status not determined)	Diarrhea, other complaints	Fecal samples	LM, confirmed by PCR, sequence analysis	17.4% (310/1,779) with diarrhea; 16.8% (112/667) without diarrhea	[18]
	Uganda	HIV+ children;HIV– children	Persistent diarrhea, dehydration	Fecal samples	PCR	Ovelall 32.9% (80/243); 76.9% (70/91) in HIV+; 6.6% (10/152) in HIV–	[64]
	Ethiopia	HIV+ and HIV– > 14 years	Diarrhea	Fecal samples	LM, PCR	12.3% (30/243) in HIV+; (0/29) in HIV–	[82]
	Tanzania	In patients HIV+ adults; HIV+ and HIV– children	Chronic diarrhea, other complaints	Fecal samples	LM, TEM	3.5% (3/86) adults; 3.4% (2/59) children with diarrhea; 20% (4/20) control children	[77]
	Zambia	HIV+	Chronic diarrhea	Fecal samples	LM	23% (16/69)	[100]
	Zimbabwe	HIV+	Persistent diarrhea	Fecal samples	LM	10% (13/129)	[57]
	Zimbabwe	HIV+	Diarrhea	Formalin-fixed fecal samples	PCR	46% (34/74)	[101]
	Zimbabwe	HIV+	Diarrhea	Fecal samples	LM, PCR	18% (10/55) by LM; 51% (28/55) by PCR	[50]
	Zimbabwe	HIV−	Diarrhea, other gastrointestinal complaints	Fecal samples	LM, confirmed by PCR	33% (2/6)	[61]
	South Africa	Hospital patients, HIV+ and HIV–; school children	Diarrhea	Fecal samples	LM, PCR-RFLP, confirmed by RT-PCR	12.9% (33/255) in hospital patients; 4.5% (3/67) of school children; 21.6% in HIV+ and 9% in HIV– with diarrhea	[54]

	China	Children	Diarrhea	Fecal samples	PCR	22.5% (9/40)	[30]
	India	HIV+	Diarrhea	Fecal samples	LM	2.5% (3/120)	[55]
	Thailand	HIV+	Chronic diarrhea	Fecal samples	LM, TEM	27.2% (18/66)	[58]
	Thailand	HIV+	Diarrhea	Fecal samples	LM, confirmed by TEM	11% (32/288)	[59]
	Thailand	HIV+ children; HIV– children	Diarrhea	Fecal samples	LM confirmed by TEM	25.3% (24/95) in HIV+; 14.9% (13/87) in HIV–	[65]
Asia	Thailand	HIV+ and HIV– children from an orphanage	Asymptomatic	Fecal samples	LM, confirmed by TEM	Overall 4.1% (12/290); 2.6% (1/39) in HIV+; 4.4% (11/251) in HIV–	[5]
	Thailand	HIV+ adults	NA	Fecal samples	LM, PCR	5.6% (5/90)	[102]
	Thailand	73 HIV+, 463 HIV−; children (<11 years) and child care workers	533 asymptomatic and 7 patients with diarrhea.	Fecal samples	LM, PCR	1.3%−6.5% overall 13.9% (75/540)	[19]
	Vietnam	HIV+	NA	Fecal samples	LM, RT-PCR, sequence analysis	7.1% (3/42)	[76]
	Australia	HIV+	Diarrhea and other gastrointestinal complaints	Duodenal biopsies	LM, confirmed by EM	30% (33/109) with diarrhea; 1.4% (1/71) without diarrhea	[39]

Oceania	Australia	HIV+	Diarrhea	Fecal samples	LM	3.6% (5/139)	[103]

LM: light microscopy, EM: electron microscopy, TEM: transmission electron microscopy, IFAT-MAb: indirect immunofluorescent assay with monoclonal antibodies, NA: not available, TB: tuberculosis, Pts: patients.

**Table 2:** Risk factors associated with *Enterocytozoon bieneusi* infection.

Risk factor	Reference
HIV/AIDS	[45, 64, 82, 86, 104]
Low CD4+ T cells count	[86]
Younger age	[18, 45, 64]
Person-to-person transmission	[5, 19, 50, 54]
Injection drugs	[86]
Living in rural areas: contact with animals	[50]
Contact with cow dung	[50]
Contact with horses	[84]
Contacts with duck or chicken droppings	[48]
Having been stung by bees, hornets, or wasps	[84]
Food: watermelon consumption	[48]
Water	
Consumption of nonpiped water	[50]
Hot tub, spa, and occupational contact with water	[86]
Contact with ground, surface, ditch, and crop irrigation water sources	[33, 105–107]
Poor sanitation	
Lack of flush toilet	[48]
Lack of garbage collection	[48]
Lack of running water	[48]

**Table 3:** Distribution of *Enterocytozoon bieneusi* genotypes in humans.

Continent	Country	Year	Patient source	Genotype (synonyms)/no. of isolates	Reference
	Peru	2003	HIV+	A (Peru1)/82, Type IV (K, Peru2, BEB5, BEB5-var, CMITS1, PtEb III)/41, Peru3/2, EbpC (E, WL13, Peru4, WL17)/4, WL11 (Peru5)/8, Peru6 (PtEb I, PtEb VII)/1, Peru7/18, Peru8/10, D (WL8, Peru9, PigEBITS9, PtEb VI, CEbc)/ 24, Peru10/6, Peru11 (Peru12)/16.	[56]
America	Peru	2000–2002	HIV+	A (Peru1)/35, Peru3/1, Peru7/8, Peru8/4, Peru11 (Peru12)/ 6, D (WL8, Peru9, PigEBITS9, PtEb VI,CEbc)/9, EbpC (E, WL13, Peru4, WL17)/1, Peru10/3, Type IV (K, Peru2, BEB5, BEB5-var, CMITS1, PtEb III)/ 18, WL11(Peru5)/3, Peru6 (PtEb I, PtEb VII)/1.	[48]
	Peru	2007	Children apparently immunocompetent	A (Peru1), Type IV (K, Peru2), Peru3, EbpC (Peru4), WL11(Peru5), Peru6 (PtEb I, PtEb VII), Peru7, Peru8, D (WL8, Peru9, PigEBITS9, PtEb VI, CEbc), Peru10, Peru11 (Peru12), Peru13, WL15 (Peru14), Peru15, Peru16, Peru17	[75]

	Portugal	1999- 2009	HIV+, HIV−; Adults and children	Type IV (K, Peru2, BEB5, BEB5-var, CMITS1, PtEb III)/18, Peru6 (PtEb I, PtEb VII)/14, D (WL8, Peru9, PigEBITS9, PtEb VI, CEbc)/6, A (Peru1)/4, C (Type II)/3, PtEb II/3.	[45]
	Spain	2009	HIV−; renal transplant recipients	D (WL8, Peru9, PigEBITS9, PtEb VI, CEbc)/2	[108]
	France	1998	HIV+; immunocompromised HIV−	B (Type I)/51 HIV+, C (Type II)/6 HIV+, 2 HIV–, Type III/3 HIV+, Type IV (K, Peru2, BEB5, BEB5-var, CMITS1, PtEb III)/3 HIV+	[109]
	France	2001	HIV+; HIV−	B (Type I)/66 HIV+, C (Type II)/9 HIV+, 8 immunocompromised HIV–, and 1 immunocompetent ), Type III/3 HIV+, Type IV (K, Peru2, BEB5, BEB5-var, CMITS1, PtEb III)/9 HIV+, 2 immunocompromised HIV–, 1 immunocompetent, Type V/1 HIV+	[92]
	Germany	1993	HIV+	A(Peru1)/3, B (Type I)/3, C (Type II)/2	[94]
Europe	Germany	2001	Human patients	C (Type II)/1, Q/1	[95]
	Switzerland	2000	Human patients	Q	[110]
	UK	1992–1995; 2002	HIV+	B (Type I)/11, D (WL8, Peru9, PigEBITS9, PtEb VI,CEbc)/1, Type IV (K, Peru2, BEB5, BEB5-var, CMITS1, PtEb III)/1	[111]
	The Netherlands	1996–2007		A(Peru1)/1, B (Type I)/4, C (Type II)/5, S7/1, S8/1, S9/1, D (WL8, Peru9, PigEBITS9, PtEb VI,CEbc)/2, Type IV (K)/5,	[112]
	Czech Republic	2008-2009	HIV−	EbpA/10, CZ3/4, PigEBITS5/3, BFRmr2/2, BEB4/2, CZ1/1, CZ2/1	[20]
	Russia		HIV+ patients	D (WL8, Peru9, PigEBITS9, PtEb VI, CEbc)/2	[87]

	Niger	2007	HIV+ (227 adults, 1 child)	A (Peru1)/10, D (WL8, Peru9, PigEBITS9, PtEb VI,CEbc)/1, Type IV (K, Peru2, BEB5, BEB5-var, CMITS1, PtEb III)/1, CAF1 (PEbE)/2, NIA1/3	[76]
	Nigeria	2011	Children with and without diarrhea	D (WL8, Peru9, PigEBITS9, PtEb VI, CEbc)/2, Type IV (K, Peru2, BEB5, BEB5-var, CMITS1, PtEb III)/1, new genotype (similar to genotype K (with two nucleotide substitutions)/1	[67]
	Nigeria	2012	HIV+	B/1, new genotype (similar to P, Type IV, UG2145; Peru3, PtEb IV, PtEb V)/2	[68]
Africa	Cameroon	2006	HIV+	Type IV (K, Peru2, BEB5, BEB5-var, CMITS1, PtEb III)/4	[55]
	Cameroon	2007	Villagers <1 to 80 years; 0.5% (4/758) HIV+	A (Peru1)/8, B (Type I)/3, D (WL8, Peru9, PigEBITS9, PtEb VI, CEbc)/3, Type IV (K, Peru2, BEB5, BEB5-var, CMITS1, PtEb III)/1, CAF4/5	[99]
	Gabon	2007	HIV+ > 16 years	A (Peru1)/1, D (WL8, Peru9, PigEBITS9, PtEb VI, CEbc)/1, TypeIV (K)/4, CAF1 (PEbE)/3, CAF2/1, CAF3/1, CAF4/4	[99]
	Uganda	2002	Children (immune status not determined)	Type IV (K, Peru2, BEB5, BEB5-var, CMITS1, PtEb III)/6, UG2145/1	[18]
	Malawi	2003-2004		Peru8/1, UG2145/1, S1/2, S2/11, S3/2, S4/1, S5/4, S6/2, D (WL8, Peru9, PigEBITS9, PtEb VI, CEbc)/4, Type IV (K, Peru2, BEB5, BEB5-var, CMITS1, PtEb III)/8	[112]

Asia	China	2011	Children with diarrhea	I (BEB2, CEbE)/3, J (BEB1,CEbB, PtEb X)/3, CHN1/5, CHN2/2, CHN3/4, CHN4/3	[30]
	Thailand	2005	HIV+ and HIV− children from an orphanage	A (Peru1)/10.	[5]
	Thailand	2006	HIV+ (33 adults)	D (WL8, Peru9, PigEBITS9, PtEb VI, CEbc)/12, EbpC (E, WL13, Peru4, WL17)/5, O/1, R/1, S/4, T/1, U/1, V/1, W/1, Peru 11 (Peru12)/2, PigEBITS7/4.	[113]
	Thailand	2005	Asymptomatic adults (5) and children (2)	A (Peru1)/7	[114]
	Thailand	2005		D (WL8, Peru9, PigEBITS9, PtEb VI, CEbc)/5	[102]
	Thailand	2003-2004	HIV+ and HIV− children from an orphanage	A (Peru1)/75	[19]
	Vietnam	2007	HIV+	D (WL8, Peru9, PigEBITS9, PtEb VI,CEbc)/1, EbpC (E, WL13, Peru4, WL17)/1, HAN1/1	[76]

Oceania	Australia	2005–2007	HIV+ (men) with diarrhea; CD4+ T-cell counts < 100 cells mm^−3^	B (Type I)/29	[115]

**Table 4:** *Enterocytozoon bieneusi* zoonotic genotypes.

Genotype designation	Hosts	Reference(s)
A	Humans, baboons and birds.	[19, 48, 56, 75, 76, 94, 99, 112–114, 116, 117]
D (PigITS9, WL8, Peru9, PtEbVI, CEbC)	Humans, macaque, baboons, pigs, cattle, horse, dogs, fox, raccoon, beaver, muskrat, and falcons	[24, 26, 29, 56, 75, 76, 87, 89, 99, 102, 108, 111, 112, 118, 119]
CAF1 (PebE)	Humans and pigs.	[76, 99, 120]
EbpC (E,WL13, WL17, Peru4)	Humans, pigs, beaver, otter, muskrat, raccoon, and fox.	[2, 21, 24, 28, 48, 56, 75, 76, 110, 113, 114, 116, 121]
Peru16	Humans and guinea pigs	[75]
Peru10	Humans and cats	[48, 56, 75, 122]
Type IV ( K, Peru2, PtEbIII, BEB5, BEB5-var)	Humans, cattle, cats, and dogs.	[18, 25–27, 48, 55, 56, 75, 76, 92, 95, 99, 109, 111, 112, 122–124]
WL11 (Peru5)	Humans, dogs, fox, and cats	[24, 48, 56, 75, 122, 123]
O	Humans and pigs.	[28, 95, 113, 114]
PigEBITS7	Humans and pigs.	[113, 119]
Peru6 (PtEbI, PtEbVII)	Humans, cattle, dogs, and birds	[26, 45, 48, 56, 75, 125, 126]
WL15 (WL16, Peru14)	Humans, beaver, fox, muskrat, and raccoon	[24, 56, 75]
EbpA (F)	Humans, cattle, pig, and birds	[20, 28, 95, 116, 119, 121, 127, 128]
I (BEB2, CebE)	Humans and cattle	[25, 30, 95, 124, 126, 128–130]
J (BEB1, CebB, PtEb X)	Humans, cattle, and birds	[25, 26, 30, 95, 124, 126, 128–131]
Peru7	Humans and baboons	[48, 56, 75, 117]
Peru11 (Peru12)	Humans and baboons	[48, 56, 75, 113, 117]
BEB4	Humans and cattle.	[20, 25, 29, 126, 130]
PigITS5 (PebA)	Humans and pigs.	[20, 116, 119, 120]
BFRmr2	Humans and pigs.	[20, 28]
CHN1	Humans, cattle and pigs.	[30]
CHN3	Humans and cattle	[30]
CHN4	Humans and cattle	[30]
PtEb II	Humans and birds	[45, 125]
